# Cell Volume and Sodium Content in Rat Kidney Collecting Duct Principal Cells During Hypotonic Shock

**DOI:** 10.1155/2008/420963

**Published:** 2008-07-27

**Authors:** Evgeny I. Solenov

**Affiliations:** Institute of Cytology and Genetics, Siberian Branch of Russian Academy of Sciences, Novosibirsk State University, Novosibirsk 630090, Russia

## Abstract

The purpose of this study was to investigate the time course of the volume-regulatory response and intracellular sodium concentration ([Na^+^]_i_) in the principal cells of rat kidney outer medulla collecting duct (OMCD) epithelia during acute swelling in hypotonic medium. Hypotonic shock was created by PBS diluted with 50% of water. Changes in cell volume were measured with calcein quenching method. Intracellular sodium concentration was studied with fluorescence dye Sodium Green. Principal cells of microdissected OMCD fragments swelled very fast. The characteristic time of swelling (*τ*_1_) was 0.65 ± 0.05 seconds, and the volume increased more than 60% (92.9 ± 5.6 and 151.3 ± 9.8 *μ*m^3^ control and peak volumes correspondently, *P* < .01). After cell volume reached the peak of swelling, the RVD began without lag period. The characteristic time of volume decreasing to new steady-state level (*τ*_2_) was 8.9 ± 1.1 seconds. In hypoosmotic medium, cell volume stabilized on higher level in comparison with control (110.3 ± 8.3 *μ*m^3^, *P* < .01). After restoration of the medium osmolality to normotonic, cell volume stabilized on significantly low level in comparison with control level (71.4 ± 6.1 *μ*m^3^, *P* < .01). During the hypoosmotic shock, [Na^+^]_i_ decreased from control level in isotonic PBS to the low level in hypoosmotic solution (27.7 ± 1.4 and 5.8 ± 0.23 mM, *P* < .01). Calculation of sodium content per cell has shown the significant sodium entry into the cells, which caused a temporary increase correlated with the peak of cell volume caused by swelling. The conclusion is made that in our model of hypoosmotic shock, swelling activates transporters with high permeability for Na^+^ that provides sodium flux into the cells.

## 1. Introduction

Kidney epithelium in collecting duct contacts with hypotonic fluid which osmolality varies significantly and the cells continually expose to osmotic stress. Alterations in luminal osmolality would influence cell volume. To avoid damage and perform their functions, principal cells clearly require an effective cell volume regulation mechanism. These processes, termed regulatory volume decrease (RVD), occur through activation of specific transporters in the plasma membrane that mediate net fluxes of osmotically active molecules. Despite its importance, very little is known about cell-volume regulation inkidney outer medulla collecting duct (OMCD) cells.

Cells recruit special mechanisms to correct acute swelling in hypotonic medium [1, 2]. Cell swelling has the effect of increasing K^+^ efflux, this occurs through channels distinct from those responsible for basal K^+^ conductance. Obviously, both K^+^ and Cl^−^ concentrations play key roles in governing these volume-regulatory responses. The predominant pathway for RVD is the opening of K^+^ channels which are usually large conductance [3, 4]. Little is known about the molecular basis of the anion conductance. These transporters can be quite nonselective and can include more than one type of channels [5]. In cells containing Na^+^ channels like collecting duct principal cells, Na^+^ fluxes can influence the volume-regulatory response but the role of intracellular sodium in RVD is almost unstudied. From another hand, the mechanism by which cells sense changes in cell volume and activates the appropriate transporters remains unknown. It is important to study the changes of cell volume and [Na^+^]_i_ during the process of RVD for better understanding of the mechanisms involved in this reaction. The current study was undertaken to investigate the time course of changes in cell volume and sodium concentration during the volume-regulatory response after acute swelling in hypotonic medium.

## 2. Methods

### 2.1. Animals

Wistar rats weighting 150–200 g (Breeding Laboratory of Experimental Animals, Institute of Cytology and Genetics, Novosibirsk, Russia) were kept in individual cages and received standard diet. Before the experiments, the rats were anaesthetized with pentobarbital (50 mg/kg i.p.), the kidneys were extracted and placed in ice-cold PBS, and a suspension of collecting duct fragments was prepared.

### 2.2. Perfusion Chamber and Microscopy

A superfusion chamber was constructed as an acrylic block mounted on the objective of the upright microscope (water immersion 65 magnification, numerical aperture 1.1) [6]. The perfusate flow rate was generally 20 mL/min, which gave complete solution exchange in under 200 milliseconds. Fluorescence measurements of cell volume were performed as it was described [6, 7]. Fragments of outer medullary collecting duct (OMCD) on glass plate were loaded with calcein by incubation with calcein-AM (Molecular Probes Inc., Ore, USA) (5.0 *μ*M) for 25 minutes at 23°C. The glass plate bearing the fragments of the OMCD was positioned on the stage of a microscope (LOMO-R8, St. Petersburg, Russia). Calcein fluorescence was measured continuously with halogen light source, calcein filter set (480 nm excitation, 490 nm dichroic mirror, 535 nm emission), photomultiplier detector has pinhole diaphragm to select the cells of interest at the end of fragment where cells expose apical surface to bath solution, and 14-bit analog-to-digital converter. The rate of data acquisition was 200 milliseconds. In these experiments, [Na^+^]i was measured using the fluorescent indicator Sodium Green. Loading conditions and filter set for fluorescence measurements were the same as for calcein.

### 2.3. Collecting Duct Fragments

Tissue from the outer medulla zone was cut from the isolated kidneys and squeezed through a needle (0.45 mm i.d.) in the ice-cold calcium-free PBS. The resulting suspension was filtered through nylon mesh, diluted 10 times with Eagle MEM culture medium and centrifuged (100 g, 10 minutes, 4°C). Sediment containing the tubules was diluted with culture medium to an appropriate concentration of about 10 fragments per microliter. This suspension was used in experiments as a preparation of OMCD fragments [6].

### 2.4. Solutions

The solutions used were based on PBS (137 mM NaCl, 4.7 mM Na_2_HPO_4_, 2.7 mM KCl, 1.5 mM KH_2_HPO_4_, 0.5 mM MgCl_2_, 0.05 mM CaCl_2_, 280 mOsm/l, pH = 7, 2–7, 1) and contained 1.0 mg/mL glucose. This solution was chosen so as to be able to degas it without affecting its pH. To create osmotic challenges, bath solutions were changed from normal PBS to PBS diluted with distilled water (1:1). Isotonic solutions with decreased [Na^+^]_o_ were prepared by substitution part of sodium in PBS by NMDG (n-methyl-D-glucamine, Sigma-Aldrich, Moscow, Russia). For measurements of sodium efflux in isotonic medium was used an extracellular isotonic solution with 69 mM [Na^+^]_o_.

### 2.5. Calibration of Cell Volume Measurements with Calcein Fluorescence

**Figure 1:**
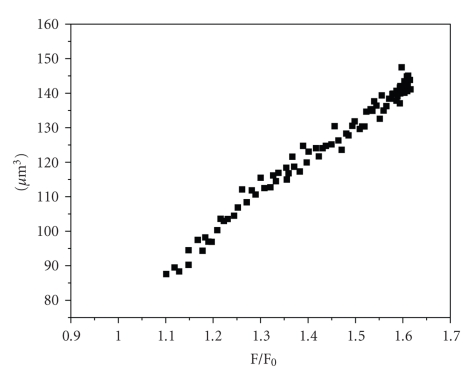
Calibration curve for calcein fluorescence quenching method. Absciss: relative fluorescence (F/F_0_). F_0_: fluorescence of intact cells in isotonic PBS. F: current fluorescence. Ordinate: absolute cell volume calculated from cells images.

Fluorescent measurements were calibrated plotting the relative values of fluorescence as an *X* value, against synchronized data of microscopic image analysis, obtained by the method published before [7]. A perfusion chamber, described above, was mounted on the objective of the microscope (water immersion 65 magnification, numerical aperture 1.1, 1.6 magnification photo adapter). The glass plate bearing the fragments of the OMCD was positioned on the stage of a microscope for acquisition of transmitted light images by a CCD camera. Twelve-bit monochrome images were captured at a rate of 15 frames per second, stored on a personal computer. The result was linear calibration plot (see Figure 1). Cell volume was calculated from measurements of calcein fluorescence according this calibration.

### 2.6. Calibration of [Na^+^]_i_

**Figure 2:**
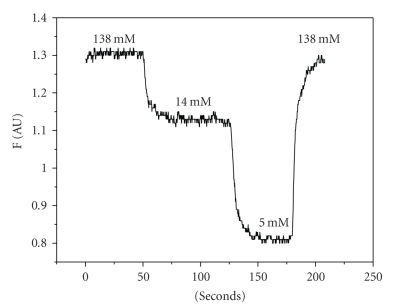
Calibration curve for Sodium Green fluorescence (representative curve). Absciss: time (seconds). Ordinate: intensity of fluorescence (F) in arbitrary units.

In vivocalibration of Sodium Green was accomplished by exposing the fragments to various extracellular [Na^+^] ([Na^+^]_o_) in the presence of 0.1 U/*μ*l Nystatin. The solutions with various [Na^+^]_o_ were prepared by substitution part of sodium in PBS by NMDG. For calibration were used solutions with 138, 14, and 5 mM [Na^+^]_o_. A calibration was performed at the end of each experiment (see Figure 2).

### 2.7. Statistical Analysis

Data are expressed as means ± SEM. Statistical discriminations were performed with Student's unpaired *t*-test. Values of *P* < .05 were considered as significant.

## 3. Results and Discussion

**Figure 3:**
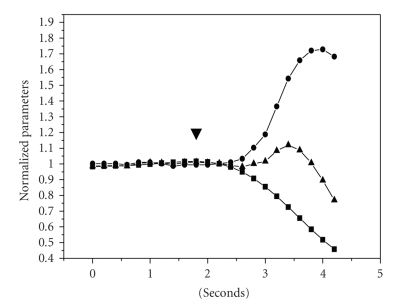
Synchronized time courses of cell volume and [Na^+^]_i_ in rat kidney OMCD principal cells. ▾: gradient signal from the valve: switch from normal PBS [Na^+^]_i_ 280 mM to hypotonic 0.5 PBS [Na^+^]_i_ 140 mM. Absciss: time (seconds). Ordinate: (•) cell volume (*μ*m^3^). (▪) intracellular sodium concentration [Na^+^]_i_ (mM). (▴) calculated content of intracellular sodium (×10 fmol/cell).

**Figure 4:**
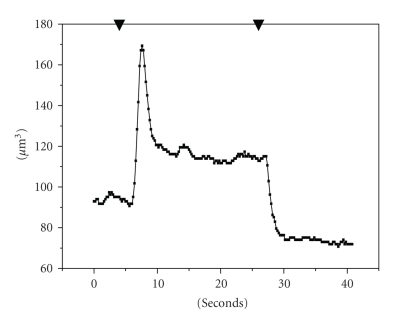
Effect of hypotonic medium on cell volume of OMCD principal cells (representative curve). Cell volumes were estimated according calcein fluorescence intensity. ▾: gradient signal from the valve: switch from normal PBS [Na^+^]_i_ 280 mM to hypotonic 0.5 PBS [Na^+^]_i_ 140 mM, and back to normal PBS. (1) control normal PBS [Na^+^]_i_ 280 mM. (2) peak of cell volume during swelling. (3) steady state in hypotonic 0.5 PBS [Na^+^]_i_ 140 mM.

Ion fluxes that appeared during cell swelling seem to be critically involved in the regulation of cell volume. However, the nature of the channels and the mechanism of their activation are poorly understood. It is also not clear to what extent this activation can explain the regulatory volume decrease. The goal of this study is to examine the time course of cell volume changes and concomitant variations in [Na^+^]_i_ after hypotonic shock. In hypotonic medium, principal cells in OMCD fragments swell very fast, and the volume increases more than 60% (see Figures 2 and 3). The evaluation of characteristic time of swelling (*τ*_1_) gives the value 0.65 ± 0.05 seconds (*n* = 12), which is in a good agreement with our previous results. In our experiments, basolateral and apical surfaces of the cells on the end of microdissected fragment are in contact with medium. Fast swelling of the principal cells is the consequence of the high water permeability determined by the water channels that are expressed in basolateral and apical membranes [8, 9]. It was shown that abundance of water channels correlates with faster RVD [10, 11]. Probably the water channels are part of the mechanism, which is activated in cell volume decrease response. After cell volume reaches the peak of swelling, the RVD begins without lag period. The kinetics of this process is slower than swelling, and the characteristic time of volume decreasing to steady-state level (*τ*_2_) is 8.9 ± 1.1 seconds (*n* = 12) (see Figure 4). The published data show that cells in cell lines derived from kidney epithelia, RCCD, COS-7, A6 have a significantly slower rate of RVD, and *τ*_2_ is at least one order more [11–13]. There is a discrepancy of results obtained on microdissected fragments of OMCD and published data obtained on cell lines. Most probably the difference is due to the loss of some ion transporters, which could not be activated by hypoosmotic shock in cultured cells. After restoration of the medium osmolality to normotonic, the cells shrink and the volume stabilizes on significantly lower level in comparison with control level (see Figure 4). This decrease probably is due to the loss of cellular osmolites during RVD. Salt withdrawal procedure could result in responses that depend on the type of cation removed. In this study, we reduced the osmolality by diluting PBS with water or removing NaCl from the bath. The procedure causes drastic changes in ionic strength that could interfere with the mechanisms involved in volume recovery.

**Figure 5:**
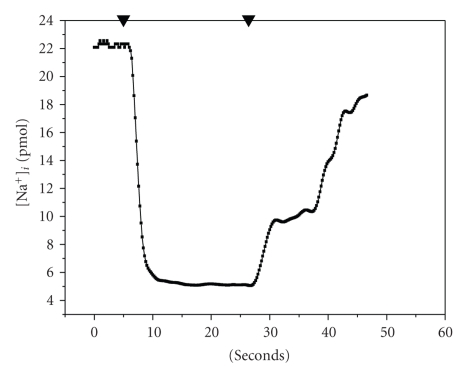
Time course of intracellular sodium concentration in OMCD principal cells. Effect of hypotonic medium (representative curve). ▾: gradient signal from the valve (same as in Figure 4). (1) control normal PBS [Na^+^]_i_ 280 mM. (2) steady state in hypotonic 0.5 PBS [Na^+^]_i_ 140 mM.

Hypotonic medium causes decrease of [Na^+^]_i_ and stabilization on the low level (see Figures 2 and 5). The established level of [Na^+^]_i_ during the hypotonic shock is the result of interaction of three main processes: dilution of osmolites with water that enters the cell, activity of Na, K-ATPase, and ion flux into the cell through channels activated by swelling, which could be permeable for sodium. Comparing the rates of normalized cell volume and [Na^+^]_i_ shows that cell volume grows about two times faster than [Na^+^]_i_ decreases (0.72 ± 0.05 and −0.30 ± 0.02 s^−1^ correspondingly, *P* < .01). This could be explained if during swelling water enters the cell accompanied by sodium. To block cell swelling and water entry, we created the transmembrane sodium gradient with normotonic PBS where 69 mM of Na^+^ was substituted by NMDG. In the absence of cell swelling with virtually zero water entry, the *τ* of [Na^+^]_i_ decrease is significantly faster in comparison with the same parameter in hypoosmotic shock (2.6 ± 0.26 and 8.9 ± 0.98 seconds, * n* = 8 and *n* = 12, correspondently, *P* < .01). These results could be accepted as evaluation of sodium efflux through Na, K-ATPase. The evaluation of sodium content per cell, made by multiplication of synchronized measurements of calcein and Sodium Green fluorescence, shows that peak of cell volume correlates with the temporary increase of this calculated parameter (see Figures 2 and 5). These results indicate that the water comes into cell during the swelling together with sodium. This could happen if swelling activated several types of channels which could pass ions with high conductivity and probably there are some channels which could pass water. Probably, this sodium flux aggravates the cell swelling in the hypotonic medium if [Na^+^]_o_ is higher than [Na^+^]_i_ but during the phase of cell volume decreasing these channels being active could promote the outcome of potassium from the cell. The outcome of the ions, mainly potassium, should be facilitated by partial depolarization which could happened in these cells during swelling [14].

**Figure 6:**
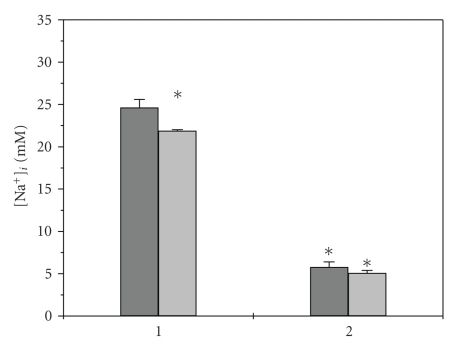
Intracellular sodium concentration in OMCD principal cells. Effect of amiloride. Gray columns: control, light gray columns: 10^−5^ M amiloride. (1) control normal PBS [Na^+^]_i_ 280 mM. (2) steady state in hypotonic 0.5 PBS [Na^+^]_i_ 140 mM. (*): *P* < .05 difference with intact control.

One of the most probable transporters active in normal conditions and which probably takes a part in volume regulating response is epithelial sodium channel (ENaC). This channel is expressed in apical membranes of principal cells, and there are data that indicate that ENaC could be involved in RVD [15, 16]. To evaluate the putative influence of ENaC on [Na^+^]_i_ during hypoosmotic challenge, we blocked it with amiloride. The addition of amiloride to the concentration of 10^−5^ M in bathing solutions caused decrease of control steady-state level of [Na^+^]_i_ (see Figure 6), but did not influence this parameter in hypotonic medium and after returning the normotonic PBS. Amiloride did not influence significantly the relative swelling during hypoosmotic challenge (0.65 ± 0.01 control, 0.77 ± 0.05 amiloride, NS). The ineffectiveness of amiloride means that contribution of ENaC in sodium entry in our model of hypotonic shock is negligible and cannot be detected in our experiments.

The results of these experiments indicate that in principal cells hypoosmotic shock causes activation of the transporters, which have high permeability for water and probably provide sodium and potassium transport across cell membrane in dependence of the ion electrochemical gradient which is forming during swelling.
